# Comparison of the effectiveness and safety of treatment of incomplete second trimester abortion with misoprostol provided by midwives and physicians: a randomised, controlled, equivalence trial in Uganda

**DOI:** 10.1016/S2214-109X(22)00312-6

**Published:** 2022-08-26

**Authors:** Susan Atuhairwe, Josaphat Byamugisha, Othman Kakaire, Claudia Hanson, Amanda Cleeve, Marie Klingberg-Allvin, Nazarius Mbona Tumwesigye, Kristina Gemzell-Danielsson

**Affiliations:** a Department of Obstetrics and Gynecology, Makerere University College of Health Sciences, Kampala, Uganda; b Department of Epidemiology and Biostatistics, School of Public Health, Makerere University College of Health Sciences, Kampala, Uganda; c Department of Public Health Sciences, Karolinska Institutet, Stockholm, Sweden; d Department of Women and Children's Health, Karolinska Institutet, Stockholm, Sweden

## Abstract

**Background:**

To address the knowledge gaps in the provision of post-abortion care by midwives for women in the second trimester, we investigated the effectiveness and safety of treatment for incomplete second trimester abortion with misoprostol, comparing care provision by midwives with that provided by physicians in Uganda.

**Methods:**

Our multicentre, randomised, controlled, equivalence trial undertaken in 14 health facilities in Uganda recruited women with incomplete abortion of uterine size 13–18 weeks. We randomly assigned (1:1) women to clinical assessment and treatment by either midwife or physician. The randomisation sequence was computer generated, in blocks of four to 12, and stratified for study site. Participants received sublingual misoprostol (400 μg once every 3 h for up to five doses). The study was not concealed from the health-care providers and study participants. Primary outcome was complete abortion within 24 h that did not require surgical evacuation. Analysis was per-protocol and intention to treat; the intention-to-treat population consisted of women who were randomised, received at least one dose of misoprostol, and reported primary outcome data, and the per-protocol population excluded women with unexplained discontinuation of treatment. We used generalised mixed-effects models to obtain the risk difference. The predefined equivalence range was –5% to 5%. The trial was registered at ClinicalTrials.gov, NCT03622073.

**Findings:**

Between Aug 14, 2018, and Nov 16, 2021, 1191 eligible women were randomly assigned to each group (593 women to the midwife group and 598 to the physician group). 1164 women were included in the per-protocol analysis, and 530 (92%) of 577 women in the midwife group and 553 (94%) of 587 women in the physician group had a complete abortion within 24 h. The model-based risk difference for the midwife versus physician group was –2·3% (95% CI –4·4 to –0·3), and within our predefined equivalence range (–5% to 5%). Two women in the midwife group received blood transfusion.

**Interpretation:**

Clinical assessment and treatment of second trimester incomplete abortion with misoprostol provided by midwives was equally effective and safe as when provided by physicians. In low-income settings, inclusion of midwives in the medical management of uncomplicated second trimester incomplete abortion has potential to increase women's access to safe post-abortion care.

**Funding:**

Swedish Research Council and THRiVE-2.

## Introduction

Unsafe abortion in middle-income and low-income countries is a public health concern, with nearly 23 000 women dying from unsafe procedures annually.^1^ Sub-Saharan Africa accounts for more than 97% of all unsafe abortions globally.^2^ Abortion-related complications, such as haemorrhage, anaemia, infection, and injury to the uterus and other internal organs,^3^, ^4^ can lead to worse outcomes when the abortion is unsafely induced versus spontaneous abortion,^5^ or in the second trimester versus first trimester.^6^ An effective intervention to manage complications and reduce abortion-related morbidity and mortality globally is post-abortion care.^7^ Post-abortion care is defined as the comprehensive treatment of women presenting after spontaneous or safely or unsafely induced abortion.^3^ Emergency treatment, counselling, family planning, other reproductive and related health services, and community–service provider partnerships constitute the five key elements of post-abortion care.^8^ In women with incomplete abortion, uterine evacuation as part of emergency treatment is critical to control bleeding and reduce risks of infection or sepsis.

Misoprostol, manual vacuum aspiration, and dilation and evacuation are uterine evacuation methods recommended by WHO.^3^ Although all three methods can be used in second trimester post-abortion care, only the first two are recommended for first trimester post-abortion care.^3^ Midwives have been shown to safely and effectively manage first trimester incomplete abortion with misoprostol and manual vacuum aspiration.^9^, ^10^, ^11^ However, there is limited evidence regarding the role midwives have in the management of second trimester incomplete abortion,^12^ and no international recommendations exist that support task sharing between physicians and midwives in this gestation period.^13^

**Research in context**

**Evidence before this study**

WHO recommends that midwives and physicians can provide care for first trimester incomplete abortion. Our trial in 2015 provided evidence that post-abortion care with misoprostol provided by midwives is equally effective and safe as when provided by physicians. We searched PubMed, Web of Science, and LISTA for articles published in English from database inception to Feb 1, 2022, with the keywords “incomplete abortion”, “post abortion care”, “second trimester”, “misoprostol”, “task sharing”, “midlevel providers”, and “non-physicians”. There was no evidence of a randomised clinical trial comparing the effectiveness and safety of the management of second trimester incomplete abortion provided by midwives with that provided by physicians.

**Added value of this study**

Our study findings show that midwives can manage uncomplicated second trimester incomplete abortions using repeat doses of misoprostol and, hence, support task sharing to midwives. Although the assumption is that most cases of post-abortion care in the second trimester are complicated and require surgical evacuation, our study suggests that 93% of women can have a complete abortion from misoprostol treatment.

**Implications of all the available evidence**

Programming for improved accessibility of post-abortion care should consider including midwives in the provision of services, not only for first trimester abortion care but also second trimester abortion care. We need efforts that focus on implementation, in which midwives are enabled and encouraged to take on this task with appropriate (pre-service and in-service) training and support so that access to services is improved while quality is sustained.

Uganda is a low-income country with a low contraceptive prevalence rate of 39% in married women, a high total fertility rate (5·4 children per woman), and 28% of married women have an unmet need for contraception.^14^ High rates of unintended pregnancies combined with restrictive abortion laws force women into unsafe abortion, which is defined as “a procedure for terminating an unintended pregnancy, carried out either by persons lacking the necessary skills or in an environment that does not conform to minimal medical standards, or both.”^3^ An estimated 130 000 women have abortion-related complications in Uganda annually,^15^ and 8% of maternal deaths are related to abortion. Harmful abortion practices, stigma, delayed health-care-seeking behaviour, and insufficient resources exacerbate post-abortion complications.^16^

Task sharing is an important strategy to enhance continuity of service delivery in countries such as Uganda where doctors and physicians are scarce.^9^ Midwives are often the key providers of post-abortion care in the first trimester, and mainly consulting physicians if a patient's condition worsens.^16^ The current Ministry of Health policy in Uganda allows both midwives and physicians to manage women with first trimester incomplete abortions, but defers management of second trimester incomplete abortion to physicians.^17^ However, there are challenges with accessing physician-only services, especially in the remote areas in which equitable service delivery is affected. Building on lessons learnt from our trial in first trimester post-abortion care,^11^ we aimed to compare the effectiveness and safety of treatment outcomes for incomplete second trimester abortion with misoprostol provided by midwives versus provision by physicians in Uganda.

## Methods

### Study design and participants

This study used a multicentre, randomised, controlled, equivalence design to compare the effectiveness and safety of second trimester incomplete abortion clinically assessed and treated with misoprostol provided by midwives with that provided by physicians. 10 months after trial commencement, we increased the number of study sites from eight to 14 to increase study participant recruitment. No other substantial change was made to the protocol.^18^ The trial and study protocol adhered to the CONSORT guidelines for reporting non-inferiority and equivalence randomised trials.^19^ We included women with symptoms and signs of incomplete abortion (eg, vaginal bleeding, uterine contractions, partial expulsion, and an open cervical os) and a uterine size of 13–18 weeks of gestation. Women were excluded if they met any of the following exclusion criteria: younger than 15 years; known allergy to misoprostol; unstable haemodynamic status (systolic blood pressure <90 mm Hg) and shock; signs of pelvic infection or sepsis, or both; previous caesarean delivery or a uterine scar; suspected extrauterine pregnancy; perforation of the uterus; injury to the surrounding organs; heavy vaginal bleeding; severe abdominal pain; cervical tear; body temperature higher than 38°C; and current molar abortion.^18^ Previous misoprostol studies for post-abortion care have used similar criteria for participant eligibility.^10^, ^11^

Post-abortion care is a key component of emergency obstetric care services in Uganda and is available from primary level health facilities up to referral hospitals.^17^ The study was implemented at 14 public health facilities, comprising four health centre IVs, eight general hospitals, and two referral hospitals, in central Uganda. All included health facilities offered comprehensive emergency obstetric care services and had both cadres of health-care providers—ie, physicians and midwives—that were requisite for trial implementation. We halted participant recruitment at four study sites prematurely because of slow enrolment at three health facilities, and the fourth was converted into a COVID treatment centre in March, 2020. Data were collected from Aug 14, 2018, to Nov 16, 2021. This study was approved by the Makerere University School of Medicine Research and Ethics Committee (rec reference 2017–016), Uganda National Council for Science and Technology (HS153ES), and administrative clearance was granted from all participating health facilities. All participants provided written informed consent.

Before trial commencement, the research team held sensitisation talks at each health facility to inform the health-care providers about the trial, obtain feedback on possible implementation improvements, and identify trainees with sufficient experience in post-abortion care (preferably practising post-abortion care providers). 183 health-care providers from the 14 study sites had a standardised 2-day training session on medical post-abortion care, and research assistants received additional training on research conduct and study procedures. The midwives’ educational background varied, from enrolled (certificate: 18 months), registered (diploma: 3 years), and dual trained (nurse-midwife: 4 years), up to degree level (certificate 4 years). The physicians comprised medical officers (doctors with 5 years of medical training), obstetrics and gynaecology residents, and obstetricians and gynaecologists. Although the trial implementation focused on physicians and midwives, we also trained clinical officers so that they could refer potential study participants to the research team. Clinical officers sometimes have the first encounter with a woman seeking care, and their engagement supported the facility research team. At the start of training, we obtained information on health-care providers’ sociodemographics, previous in-service post-abortion care trainings attended, and their self-reported confidence in using misoprostol or manual vacuum aspiration for post-abortion care generally. We also held specific on-job training for newly deployed staff at the study sites, and this training ensured that all involved staff had a standardised training in post-abortion care according to our trial protocol.

### Randomisation and masking

An independent statistician made a computer-generated blocked randomisation list stratified for each study site with a 1:1 ratio for physicians versus midwives in random blocks of four to 12. Block sizes were not disclosed to the research assistants to ensure concealment. Allocation was concealed from the research assistants using sequentially numbered sealed opaque envelopes, and research assistants opened the envelopes in consecutive order after obtaining informed consent. The study coordinators made visits once every 2 weeks to the study sites and checked adherence to the study procedures, made corrections to the filled protocols, and provided continuous support to the research assistants and health-care providers. The first author (SA) cross-checked all protocols for completeness and accuracy before data entry and provided overall supervision and support to the study coordinators and health facility research teams. The study was not concealed from the health-care providers and study participants. The allocation groups were concealed from data entrants and the data and safety monitoring board using codes.

### Procedures

Trained midwives used a predefined checklist to screen all women with abortion complications for eligibility. Non-eligible women were also registered and the reason for exclusion was specified in a logbook. Research assistants obtained informed written consent from eligible participants and allocated them to the randomly assigned groups, either the midwife (intervention) group or the physician (control) group, for clinical assessment and management. The clinical assessment comprised history taking of sociodemographic, reproductive, and medical information, and noting presenting symptoms. On physical examination, allocated health-care providers assessed vital signs (eg, pulse, blood pressure, and temperature) and uterine size, and performed a vaginal examination to check for cervical opening, bleeding, and signs of genital infections.^18^ All participants were admitted for observation and the allocated health-care providers treated participants with 400 μg misoprostol sublingually once every 3 h, not exceeding five doses or until a complete abortion was confirmed;^3^ the health-care providers assessed vital signs, presence of side-effects that were unexpected, uterine contractions, and vaginal bleeding before the subsequent dose of misoprostol was given. Once expulsion of products of conception (ie, placental tissue alone, placenta and partial fetus, or whole fetus and placenta) occurred, the allocated health-care provider diagnosed a complete abortion and an independent assessor (same cadre) verified the diagnosis. If the independent assessor diagnosed an incomplete abortion, then treatment would continue as per protocol; however, if 24 h had elapsed since treatment initiation, then it would be defined and noted down as treatment failure. All participants with incomplete abortion at 24 h after treatment initiation or with a deteriorating clinical status were managed surgically with manual vacuum aspiration or curettage by a physician. During the admission, study participants also received analgesics, prophylactic antibiotics, contraceptive counselling, and pre-discharge information on signs for possible concern (eg, heavy vaginal bleeding, increased abdominal cramps, fever, and dizziness), and were scheduled to return after 2 weeks for a follow-up visit.

### Outcomes

The primary outcome was a complete abortion without any surgical intervention within 24 h of treatment initiation. Clinical assessment of the primary outcome included physical and pelvic examination with attainment of a complete abortion based on cessation of uterine cramps and vaginal bleeding, and a closed cervical os. Secondary outcomes measured at the initial visit were time from induction (first dose of misoprostol) to completion of abortion and total dose of misoprostol given. Secondary outcomes assessed at a 2-week follow-up visit to measure safety comprised the following: excessive vaginal bleeding (soaking more than three pads in 1 h); abdominal pain (discomfort in the lower abdomen) and its intensity (measured with a visual analogue scale; minimum score of 0 representing no pain and maximum score of 10 representing most pain); unscheduled visits (participants presenting at the study site when not expected); need for additional medical treatment; misoprostol side-effects; and sepsis (body temperature higher than 38°C, pus, or offensive vaginal discharge).^18^ Trial outcomes were not changed after study commencement. Safety was also measured in occurrence of severe adverse events, such as hospitalisation for more than 48 h, blood transfusion, disability or incapacity, life-threatening sepsis, and death, and were documented in a serious adverse event form at the end of each case report form.

### Statistical analysis

We computed the sample size with the objective of establishing two-sided equivalence if both groups had a 90% complete abortion rate at 24 h within a 10% equivalence margin. At 80% power, taking into account two-sided 95% CI, set actual difference D1 range of –5% to 5%, smallest difference of 0·001, design effect of 1, and 5% level of significance, we required 566 women in each group. PASS software (version 14) was used for statistical analysis. Considering a 5% loss to follow-up, we had a sample size of 1192 women. The Data Safety Monitoring Board performed an interim analysis on 55% accrued sample in August, 2020, to assess safety, and there were no significant differences between both groups.

We entered data into EpiData (version 3.1) and analysed with Stata (version 14). We used complete-case analysis given that we had minimal missing data (<1%). We defined the intention-to-treat population as all women who were randomised, received at least one dose of misoprostol, and reported primary outcome data. The per-protocol population excluded women with unexplained discontinuation of treatment. Background characteristics of the study participants and post-abortion care providers were analysed and presented with descriptive statistics. Categorical data were presented as proportions whereas continuous data were presented as means and SDs if normally distributed, and medians and IQRs if not normally distributed. To determine differences in participants’ background characteristics between the two study groups, we used the χ^2^ test for categorical data, the Student's *t*-test for normally distributed continuous data, and the Wilcoxon rank-sum test when continuous data were not normally distributed.

Analysis of the primary outcome (complete abortion) between the midwife and physician groups was done using risk difference with 95% CIs. Equivalence was established if the risk difference lay between the predefined range of –5% and 5%. A p value of 0·05 or less was considered statistically significant. To cater for differences in performance between the sites, we used a generalised linear mixed-effects model with health-care provider as a fixed effect and health-care facility as a random effect. Further, we used the backward regression model fitting technique starting with covariates: study group, age, marital status, religion, education level, occupation, number of pregnancies, and number of livebirths. The adjusted risk difference was estimated as the predicted risk difference at the average of the covariates study group, facility, age, and number of pregnancies in the final model. We assessed for best model fit using the Bayesian information criterion from the likelihood-ratio test. There was no interaction between age and number of pregnancies occupation. Secondary outcomes included information on safety of the intervention,^18^ and were presented descriptively. We compared outcomes between the two groups using a Student's *t*-test, a χ^2^ test, or a Wilcoxon rank-sum test.

Time from first dose of misoprostol to completion of abortion was computed and analysed using standard survival analysis techniques. Participants were censored at the time of having a complete abortion or administratively censored at 24 h if no complete abortion occurred. Median time (in h), derived from Kaplan-Meier estimates of the survival function, was compared between groups with the log-rank test. We used a Cox proportional hazards model to establish factors independently associated with time to completion of abortion. The final best fit multivariable regression model, attained with the backward regression technique, contained the study group (physician *vs* midwife), education (none *vs* primary, secondary, and tertiary), and occupation (unemployed *vs* formal employment and self-employment) as covariates. There was no interaction between education and occupation. We checked for the proportional hazard assumption using Schoenfeld residuals and it was met.

The trial is registered at ClinicalTrials.gov, NCT03622073.

### Role of the funding source

The funders of the study had no role in study design, data collection, data analysis, data interpretation, or writing of the report.

## Results

**Figure 1:**
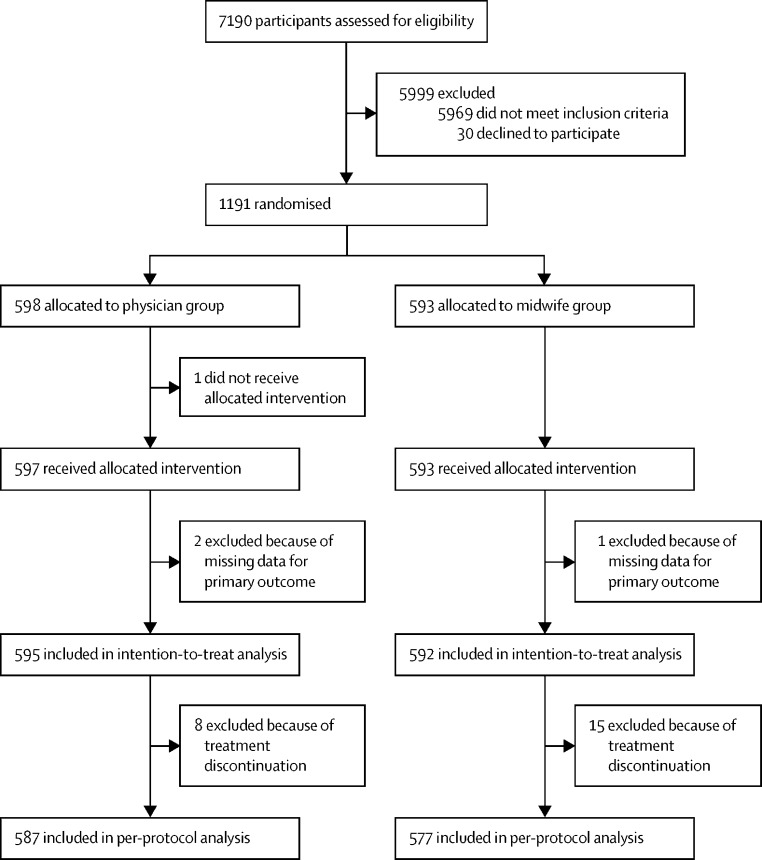
Trial profile

We screened 7190 women for eligibility between Aug 14, 2018, and Nov 16, 2021. After exclusion of 5999 women, we randomised 1191 and assigned 598 to the physician group and 593 to the midwife group (figure 1). Four study participants were excluded; one woman in the physician's group did not receive the intervention, and two women in the physician group and one woman in midwife group had incomplete primary outcome data. The two women in the physician group left the facility against medical advice; one woman returned at the 2-week follow-up and had surgical evacuation, and the other woman had an ongoing pregnancy when contacted 3 weeks later. The one woman in the midwife group had heavy bleeding and was referred to another facility because the physician was not on site to perform the evacuation.

We included 1187 women in the intention-to-treat analysis (595 in the physician group and 592 in the midwife group). We further excluded 23 women (eight in the physician group and 15 in the midwife group) who discontinued treatment for unknown reasons. These 23 women did not receive the complete five doses of misoprostol but had a surgical evacuation despite no deterioration of clinical status or reasons why the medical management was discontinued. Therefore, the per-protocol analysis of the primary outcome included 1164 women (587 in the physician group and 577 in the midwife group).

We observed no major difference between study groups with regard to the background characteristics of the participants (table 1). Participants had a mean age of 26·2 years (SD 6·0; range 15–47), and the majority were 20–24 years (33% [395/1185]). The majority were married or cohabiting (77% [914/1187]), catholic (41% [492/1187]), unemployed (47% [563/1187]), and had attained secondary education as the highest education level (44% [518/1187]). The median gestational age in weeks was 14 (IQR 13–18), the median number of pregnancies was 3 (2–4), and the median number of livebirths was 1 (0–3).

**Table 1:** Sociodemographic and reproductive history characteristics of participants (intention-to-treat population)

	**Midwife group**	**Physician group**	**Total**
**Age, years**			
Number of participants*	590	595	1185
Mean (SD)	26·0 (6·1)	26·3 (5·9)	26·2 (6·0)
**Marital status**			
Number of participants	592	595	1187
Married or cohabiting	444 (75%)	470 (79%)	914 (77%)
Single or divorced or widow	148 (25%)	125 (21%)	273 (23%)
**Religion**			
Number of participants	592	595	1187
Catholic	243 (41%)	249 (42%)	492 (41%)
Protestant	179 (30%)	191 (32%)	370 (31%)
Islam	127 (22%)	103 (17%)	230 (19%)
Other (SDA, born again)	43 (7%)	52 (9%)	95 (8%)
**Education**			
Number of participants	592	595	1187
None	16 (3%)	20 (3%)	36 (3%)
Primary	264 (45%)	246 (41%)	510 (43%)
Secondary	254 (43%)	264 (44%)	518 (44%)
Tertiary	58 (10%)	65 (11%)	123 (10%)
**Occupation**			
Number of participants	592	595	1187
Unemployed	281 (47%)	282 (47%)	563 (47%)
Formal employment	63 (11%)	79 (13%)	142 (12%)
Self-employed	248 (42%)	234 (39%)	482 (41%)
**Gestational age based on clinical examination, weeks**			
Number of participants†	589	592	1181
Mean (SD)	15·0 (1·5)	15·1 (1·6)	15·0 (1·6)
Median (IQR)	14 (13–18)	14 (13–18)	14 (13–18)
**Number of pregnancies**			
Number of participants	592	595	1187
Median (IQR)	3 (2–4)	3 (2–4)	3 (2–4)
**Parity (livebirth)**			
Number of participants	592	595	1187
Median (IQR)	1 (0–3)	2 (0–3)	1 (0–3)

Data are n (%) unless stated otherwise. SDA=Seventh-day Adventist.

* Two women in midwife group were missing data for age.

† Three women in midwife group and three women in physician group were missing data for gestational age.

The characteristics of health-care practitioners when comparing the two study groups did not differ significantly in regard to mean age, number of in-service post-abortion care trainings, and confidence in using misoprostol (table 2). Midwives were predominantly female (97% [135/139]) and physicians were predominantly male (82% [27/33]; p<0·0001). Physicians reported more confidence in using manual vacuum aspiration, to manage incomplete abortion at any gestation age, compared with midwives (91% [30/33] *vs* 48% [63/130]; p<0·0001) before trial initiation.

**Table 2:** Background characteristics of participating post-abortion care providers before study start

		**Midwife group**	**Physician group**	**Total**	**p value**
Age, years					
	Number of post-abortion care providers	138	31	169*	..
	Mean (SD)	38·1 (9·7)	36·4 (9·2)	37·8 (9·6)	0·38†
	Median (IQR)	39 (29–45)	34 (29–40)	38 (29–45)	..
Sex		..	..	..	<0·0001‡
	Number of post-abortion care providers	139	33	172	..
	Female	135 (97%)	6 (18%)	141 (82%)	..
	Male	4 (3%)	27 (82%)	31 (18%)	..
Number of in–service post-abortion care trainings					
	Number of post-abortion care providers	111	24	135§	..
	Median (IQR)	1 (0–1)	1 (0–2)	1 (0–2)	0·16¶
Confidence in using misoprostol		..	..	..	0·29‡
	Number of post-abortion care providers	135	33	168‖	..
	Yes	126 (93%)	29 (88%)	155 (92%)	..
Confidence in using manual vacuum aspiration		..	..	..	<0·0001‡
	Number of post-abortion care providers	130	33	163**	..
	Yes	63 (48%)	30 (91%)	93 (57%)	..

Data are n (%) unless stated otherwise. 174 of 183 post-abortion care providers completed the questionnaire; two were clinical officers, and not part of the randomisation groups. Therefore, the analytical sample was 172.

* One provider in the midwife group and two providers in the physician group were missing data for age.

† Student's t–test.

‡ χ^2^ test.

§ 28 providers in the midwife group and nine providers in the midwife group were missing data for confidence using manual vacuum aspiration.

¶ Wilcoxon rank-sum test.

‖ Four providers in the midwife group were missing data for confidence using misoprostol.

** Nine providers in the midwife group were missing data for confidence using manual vacuum aspiration.

1083 (93%) of 1164 women had a complete abortion (530 [92%] of 577 women in the midwife group *vs* 553 [94%] of 587 women in the physician group), with a risk difference of –2·4% (95% CI –4·4 to –0·3%) between the two groups (table 3). The adjusted risk difference in the proportion of complete abortions between the midwife group and physician group was –2·3% (95% CI –4·4% to –0·3%) and did not cross the equivalence margins of –5% to 5% (appendix p 1). In women with complete abortion, products of conception consisted of placental tissue alone in 37% (400/1081), placental tissue and partial fetus in 24% (262/1081), and whole fetus and placental tissue in 39% (419/1081; appendix p 2). All 80 women with incomplete abortion after treatment had a surgical evacuation by physicians with either manual vacuum aspiration (43 women) or dilation and evacuation (37 women appendix p 2). Two women in the midwife group received blood transfusion because of heavy vaginal bleeding and recovered well after surgical evacuation. No other serious adverse events were reported.

**Table 3:** Treatment outcomes of second trimester incomplete abortion (per-protocol population)

	**Midwife group**	**Physician group**	**Total**	Unadjusted risk difference (95% CI)*	Adjusted risk difference (95% CI)*
**Primary outcome**					
Number of participants	577	587	1164	..	..
Complete abortion	530 (92%)	553 (94%)	1083 (93%)	−2·4 (−4·4 to −0·3)	−2·3 (−4·4 to −0·3)
**Experience of side-effects**					
Number of participants	563	574	1137†	..	..
Yes	131 (23%)	136 (24%)	267 (23%)	..	..
No	432 (77%)	438 (76%)	870 (77%)	..	..
**Bleeding during treatment**					
Number of participants	565	575	1140	..	..
Less than normal menstrual bleeding	320 (57%)	320 (56%)	640 (56%)	..	..
Same as normal menstrual bleeding	206 (36%)	213 (37%)	419 (37%)	..	..
Heavier than normal menstrual bleeding	39 (7%)	42 (7%)	81 (7%)	..	..
**Days of bleeding**					
Number of participants	557	571	1128‡	..	..
Median (IQR)	3 (2–4)	3 (2–4)	3 (2–4)	..	..
Range	1–14	1–14	1–14	..	..
**Abdominal pain intensity using visual analogue scale 0–10**					
Number of participants	564	574	1138§	..	..
Median (IQR)	2 (1–4)	2 (1–4)	2 (1–4)	..	..
Range	0–10	0–10	0–10	..	..
**Pelvic infection at follow–up visit**					
Number of participants	565	575	1140	..	..
Yes	7 (1%)	8 (1%)	15 (1%)	..	..
No	558 (99%)	567 (99%)	1125 (99%)	..	..
**Unscheduled visits**					
Number of participants	565	575	1140	..	..
Yes	20 (3%)	9 (2%)	29 (2%)	..	..
No	545 (97%)	566 (98%)	1111 (98%)	..	..
**Duration of admission at health facility**					
Number of participants	577	586	1163¶	..	..
Median (IQR)	21 (12–24)	20 (12–24)	21 (12–24)	..	..

Data are n (%) unless stated otherwise. 1140 of 1164 women returned for the 2-week follow-up visit.

* Adjusted for facility, age (15–19 years *vs* 20–24 years, 25–29 years, 30–34 years, and 35–49 years), and number of pregnancies (1 *vs* 2–4, and 5–15).

† Two women in the midwife group and one woman in the physician group were missing data for experience of side-effects.

‡ Eight women in the midwife group and four women in the physician group were missing data for days of bleeding.

§ One woman in the midwife group and one woman in the physician group were missing data for abdominal pain intensity.

¶ One participant in the physician group was missing data for duration of admission.

**Figure 2:**
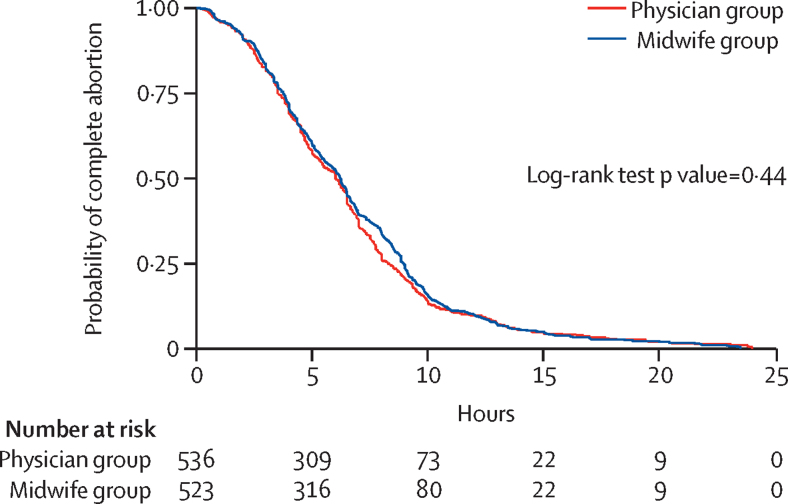
Kaplan-Meier estimates of time from first dose of misoprostol to complete abortion (n=1079; seven women in the midwife group and 17 women in the physician group were missing data for time from first dose of misoprostol to complete abortion)

In women with treatment success, the median total dose of misoprostol administered in each group was 1200 μg (IQR 800–1200), which translates to three doses. The median time from administration of the first dose of misoprostol to a complete abortion was 6·1 h (IQR 3·7–8·6). This finding was similar in the two groups (6·0 h [IQR 3·5–8·2] in the midwife group *vs* 6·2 h [3·7–8·8] in the physician group; p=0·44; figure 2). The time to complete abortion was shorter in women with tertiary level of education than in those with no education (adjusted HR 0·55 [95% CI 0·36 to 0·84]; p=0·005), and among self-employed women versus unemployed women (0·86 [0·75 to 0·99]; p=0·036). The median duration of admission at the health facility was 21 h (IQR 12–24), and was similarly distributed between the two groups (table 3).

Data for secondary outcomes collected at the 2-week follow-up visit are shown in table 3. 1140 women returned at the 2-week follow-up visit. 24 women were lost to follow-up; 12 in the midwife group and 12 in the physician group. Of the 1140, two women in the midwife group and one woman in the physician group were missing data for experience of side-effects. 267 (23%) of 1137 women reported having side-effects (table 3), with nausea, severe abdominal pain, vomiting, and diarrhoea being the most common side-effects reported (table 4). Among 1140 women, 640 (56%) had less bleeding during treatment than their normal menstrual flow, 419 (37%) had the same bleeding as the normal menstrual flow, and 81 (7%) had more than normal menstrual bleeding. The median number of days of bleeding after treatment was 3 (IQR 2–4). Of 1140 women, 15 (1%) women had a pelvic infection at the 2-week follow-up visit, but they had received prophylactic antibiotics. The median score of abdominal pain intensity was 2 (IQR 1–4). 816 (70%) of 1162 women received analgesics during treatment and 747 (65%) of 1140 swallowed analgesics after treatment (appendix p 2). There were 29 unscheduled visits (20 [3%] of 565 in the midwife group and nine [2%] of 575 in the physician group). Abdominal pain or vaginal bleeding (13/29), chills (3/29), and backache (2/29) were the most common reasons for the unscheduled visits. One woman in the physician group had a manual vacuum aspiration done at the unscheduled visit. All other secondary outcomes that depict safety of the intervention did not significantly differ between the two study groups (table 3).

**Table 4:** Types of side-effects experienced by participants treated with misoprostol

		**Midwife group**	**Physician group**	**Total**
Number of participants		563	574	1137*
Chills lasting more than 24 h		14 (2%)	16 (3%)	32 (3%)
Foul smelling vaginal discharge		7 (1%)	12 (2%)	19 (2%)
Severe abdominal pain lasting more than 24 h		21 (4%)	37 (6%)	63 (5%)
Nausea		80 (14%)	60 (10%)	149 (13%)
Vomiting		21 (4%)	33 (6%)	58 (5%)
Diarrhoea		18 (3%)	18 (3%)	36 (3%)
Fever lasting more than 24 h		7 (1%)	6 (1%)	13 (1%)
Others		18 (3%)	19 (3%)	38 (3%)
	Backache	5 (1%)	7 (1%)	12 (1%)
	Dizziness	4 (1%)	3 (1%)	7 (1%)
	Vaginal spotting	6 (1%)	3 (1%)	9 (1%)
	Mild lower abdominal pain	1 (<1%)	1 (<1%)	2 (<1%)
	Sore throat and flu	2 (<1%)	2 (<1%)	4 (<1%)
	Headache	1 (<1%)	2 (<1%)	3 (<1%)
	Vessel pain	0	1 (<1%)	1 (<1%)

Data are n (%) unless stated otherwise.

* Of the 1140 women who returned for follow-up, two women in the midwife group and one woman in the physician group were missing data for experience of side-effects.

## Discussion

Our multicentre, randomised, controlled, equivalence trial in Uganda showed that second-trimester medical treatment of incomplete abortion with misoprostol provided by midwives was equally effective and safe as when provided by physicians. This study was done across different levels within the health care system—referral hospitals, general hospitals, and health centre IVs—which makes the findings applicable to similar settings in middle-income and low-income countries. Our study is unique because, to our knowledge, it is the first randomised clinical trial to address task sharing in second trimester post-abortion care using misoprostol and shows equivalence between post-abortion care provided by midwives and by physicians. Our results have the potential to change policy and practice. Because midwives are generally higher in number, work at lower level facilities, and are more evenly distributed across the country, our findings could support policies making second trimester post-abortion care available in lower level facilities provided by midwives. The high treatment success rates of over 90% support this opportunity, as surgical evacuation was needed only in a small number of cases.

The overall proportion of women with symptoms and signs of incomplete abortion who had a complete abortion within 24 h after a maximum of five doses of misoprostol was 93%. Although first trimester post-abortion care success rates have been reported to be slightly higher (94·5–96·7%),^9^, ^10^, ^11^ our treatment success rate was more than the a priori assumed complete abortion rate of 90%.^18^ Evidence of equivalence (adjusted risk difference –2·3% [95% CI –4·4 to –0·3]) between the two groups shows that midwives can safely be included in the assessment and treatment of second-trimester incomplete abortion, with misoprostol—a service presently restricted to physicians in most parts of the world. In countries such as Uganda where physicians are few and mainly concentrated in the urban and peri-urban regions,^20^ this evidence offers an opportunity to expand equitable access to post-abortion care. Notably, midwives are often the first health-care practitioners most patients interface with at the lower level of health-care service delivery, particularly in rural areas.^16^ In Uganda, second trimester incomplete abortion is usually managed by surgical methods at a higher level of health care (from health centre IVs, to general and referral hospitals),^21^ so the introduction of misoprostol in this gestation will probably improve access to timely treatment. Nevertheless, it is crucial that health-care practitioners are appropriately trained to identify and clinically manage women with both first and second trimester incomplete abortion who would benefit from using misoprostol.^3^, ^20^ In our trial, 93% of the midwives had confidence in using misoprostol, which could be attributed to their extensive experience with first trimester post-abortion care.^7^ Yet, only 48% of midwives had confidence in using manual vacuum aspiration, highlighting the need for additional in-service post-abortion care training and support.^20^

In our study we used a clinical diagnosis of complete abortion and reported the type of expelled products of conception as either placental tissue alone, placental tissue and partial fetus, or whole fetus and placental tissue. Our approach in the diagnosis of complete abortion has been successfully used in previous post-abortion care studies.^10^, ^11^ Measurement of endometrial thickness on ultrasound scan as a proxy for diagnosing a complete abortion does not improve patient outcomes and inadvertently increases surgical procedures.^22^ Our study findings are therefore easily replicable in daily patient care points, particularly in settings with limited access to ultrasound services.

Our results indicate that 75% of the women with incomplete abortion needed only three doses of misoprostol given at a median duration of 6·1 h. WHO's 2018 guideline on medical management of abortion recommends one dose of misoprostol every 3 h for second trimester incomplete abortion at the discretion of the health-care practitioner.^23^ Our study used a maximum of five doses of misoprostol,^3^ and 23% of 1137 women reported misoprostol-related side-effects. Side-effects are likely to occur more frequently with repeated and higher total doses of misoprostol. Although a concern, side-effects are known and manageable,^24^ especially if women receive adequate information and counselling to allay any concerns and ensure treatment adherence. In our study, 23 women prematurely received a surgical evacuation. During our supervisory visits, health-care practitioners cited drivers, such as monetary gain and impatience of the staff, which we addressed during several engagement meetings with the health-care practitioners. Notably, women with tertiary education or those who were self-employed had statistically significant shorter durations of treatment, which could suggest a better understanding of the treatment procedure and better adherence to the treatment protocol given that this was an effectiveness study. Notably, self-employed women might be motivated to adhere to the treatment procedures so that they can recover quickly and return to their work for earning.

Haemorrhage and sepsis are generally known as the most frequent severe abortion-related morbidities.^3^ The rate of post-abortion pelvic infections in our study (1%) is similar to previous studies on first trimester post-abortion care.^10^ Given that the second trimester is associated with more severe abortion-related complications,^6^, ^25^ we established strategies to prevent complications, such as careful screening for study eligibility, provision of prophylactic antibiotics, and ensuring attainment of the outcome before discharge, which could have contributed to the low rate of pelvic infection. About 50% of the women bled for 2–4 days after treatment, and most described their bleeding during treatment as less than normal menstrual bleeding. All women with treatment failures had surgical evacuations performed by a physician before discharge.^18^ This approach ensured patient safety, allayed health-care practitioners’ concerns on probable outcomes, and is easily replicable in clinical practice. Although our study reports a low median pain score of 2 experienced by women during treatment, only 70% of the women received analgesics. As shown in previous studies,^6^, ^7^ there are often shortages of drugs and supplies at public health facilities, especially those with large caseloads. Our study made an effort to avail some drugs at the health facilities that lacked them but there might be occasions when women are not given the available drugs, which could depict abortion-related stigma.^7^

The strengths of this study are a large sample of 1187 women with second trimester incomplete abortions from 14 public health facilities, a randomised-controlled design, and assessment of the primary outcome at the initial visit reducing loss to follow-up. We assessed effectiveness rather than efficacy, to ensure easy replicability of study findings in clinical routine care at similar settings.

The study had some limitations. Women were randomly assigned to health-care practitioners who work closely at the post-abortion care sites and within the same environment, which could cause convergence of the outcome. Second trimester post-abortion care is typically offered by physicians rather than midwives, which could influence attitudes and technical expertise. To address this concern, all participating health-care practitioners had a preintervention standardised training and the study team visited the study sites once every 2 weeks, to address any protocol-related challenges and strengthen the internal validity of the study.

We found that misoprostol treatment of uncomplicated second trimester (13–18 weeks gestation) incomplete abortion care provided by midwives was equally effective and safe as when it was provided by physicians. The findings from this trial are of particular relevance for settings characterised by shortages of health-care providers, high patient volumes, and limited resources, including settings without access to ultrasound. In low-income settings such as Uganda, midwives have the potential to competently task share medical management of uncomplicated second trimester incomplete abortion. Task sharing could substantially increase women's access to safe and timely post-abortion care, eliminate the use of outdated methods, and reduce abortion-related morbidity and mortality. Our study findings are relevant for both practice and policy as they could influence task sharing policies and clinical practice standards and guidelines, nationally as well as internationally.

## Data sharing

The datasets used or analysed during the current study are available from the corresponding author on reasonable request. The full protocol can be accessed online.

## Declaration of interests

We declare no competing interests.
