# Supplementary material for: Comparison of the effectiveness and safety of treatment of incomplete second trimester abortion with misoprostol provided by midwives and physicians: a randomised, controlled, equivalence trial in Uganda
Source: Lancet Glob Health. 2022 Aug 26;10(10):e1505–13. doi: 10.1016/S2214-109X(22)00312-6 (PMC9605879; doi:10.1016/S2214-109X(22)00312-6)
Supplement: Supplementary appendix [file mmc1.pdf]

THE LANCET
Global Health

Supplementary appendix

This appendix formed part of the original submission and has been peer reviewed. We post it as supplied by the authors.

Supplement to: Atuhairwe S, Byamugisha J, Kakaire O, et al. Comparison of the effectiveness and safety of treatment of incomplete second trimester abortion with misoprostol provided by midwives and physicians: a randomised, controlled, equivalence trial in Uganda. *Lancet Glob Health* 2022; published online Aug 25. https://doi.org/10.1016/S2214-109X(22)00312-6.

Manuscript reference number: LANGLH-D-22-00356R

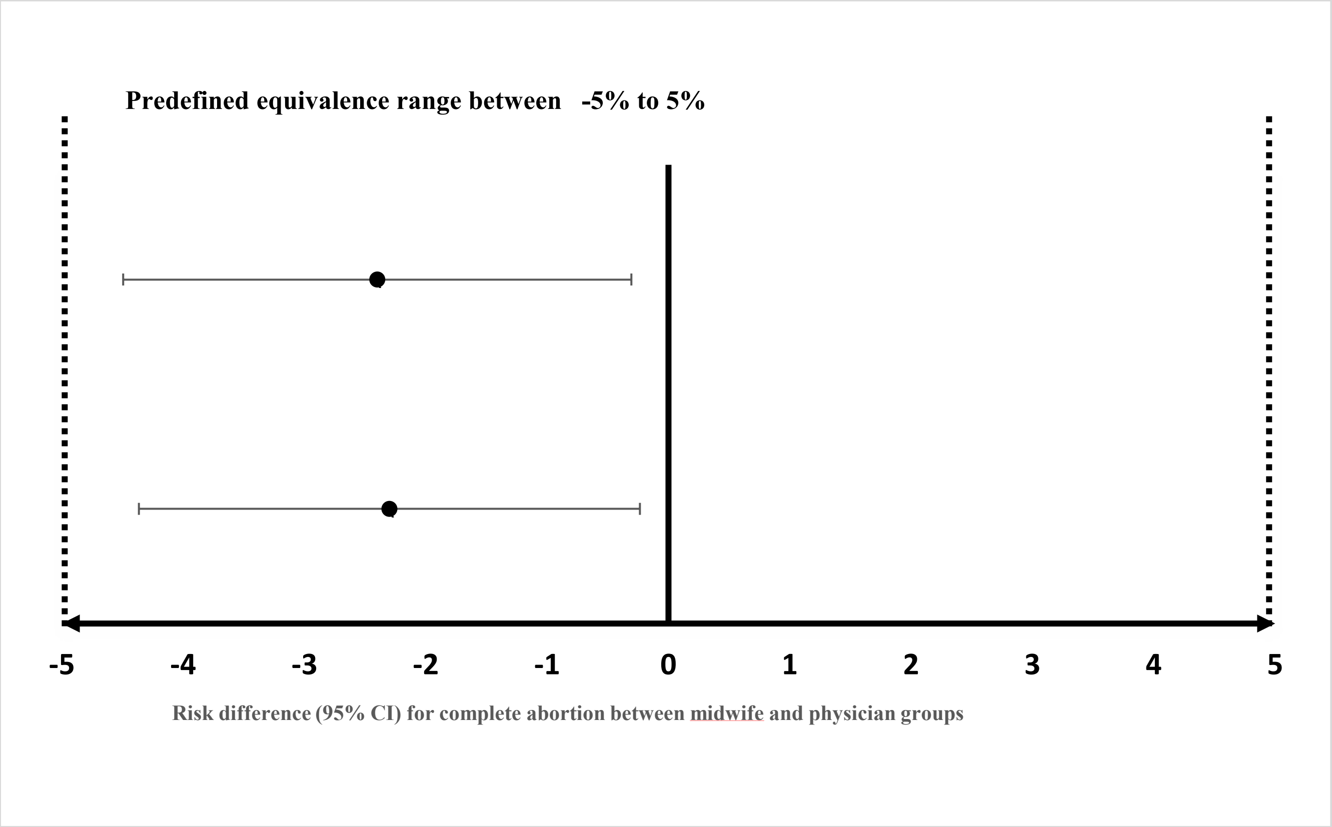

Appendix: Risk difference (95% CI) of complete abortion among midwife and physician groups and predefined equivalence range

Appendix: Types of products expelled after treatment with misoprostol and adjuvant treatment provided

	Midwife group	Physician group	Total
**Types of products expelled**			
Number of participants*	530	551	1081
Placental tissue alone	119(38%)	201(36%)	400(37%)
Placental tissue and partial fetus	118(22%)	144(26%)	262(24%)
Whole fetus and placental tissue	213(40%)	206(37%)	419(39%)
**Type of surgical evacuation**			
Number of participants[#]	47	33	80
Manual vacuum aspiration	24(51%)	19 (58%)	43 (54%)
Dilation and evacuation	23(49%)	14(42%)	37(46%)
**Received analgesics during treatment**			
Number of participants[a]	575	587	1162
Yes	410(71%)	406(69%)	816(70%)
**Swallowed analgesics after treatment**			
Number of participants	565	575	1140
Yes	371(66%)	376 (65%)	747 (65%)

Data are n (%) unless otherwise stated. *2 participants in the physician group had missing data for type of products expelled. [#]1 participant in the physician group had missing data for type of surgical evacuation. [a]2 participants in the midwife group had missing data for swallowed analgesics after treatment.
